# A locus for an auditory processing deficit and language impairment in an extended pedigree maps to 12p13.31-q14.3

**DOI:** 10.1111/j.1601-183X.2010.00583.x

**Published:** 2010-08

**Authors:** L Addis, A D Friederici, S A Kotz, B Sabisch, J Barry, N Richter, A A Ludwig, R Rübsamen, F W Albert, S Pääbo, D F Newbury, A P Monaco

**Affiliations:** † Wellcome Trust Centre for Human Genetics, University of OxfordRoosevelt Drive, Oxford OX3 7BN, UK; ‡ Max Planck Institute for Human Cognitive and Brain SciencesLeipzig, Germany; § Institute of Biology II, University of LeipzigLeipzig, Germany; ¶ Max-Planck Institute for Evolutionary AnthropologyLeipzig, Germany

**Keywords:** Auditory processing deficit, chromosome 12, language impairment, late discrimination negativity, nonword repetition

## Abstract

Despite the apparent robustness of language learning in humans, a large number of children still fail to develop appropriate language skills despite adequate means and opportunity. Most cases of language impairment have a complex etiology, with genetic and environmental influences. In contrast, we describe a three-generation German family who present with an apparently simple segregation of language impairment. Investigations of the family indicate auditory processing difficulties as a core deficit. Affected members performed poorly on a nonword repetition task and present with communication impairments. The brain activation pattern for syllable duration as measured by event-related brain potentials showed clear differences between affected family members and controls, with only affected members displaying a late discrimination negativity. In conjunction with psychoacoustic data showing deficiencies in auditory duration discrimination, the present results indicate increased processing demands in discriminating syllables of different duration. This, we argue, forms the cognitive basis of the observed language impairment in this family. Genome-wide linkage analysis showed a haplotype in the central region of chromosome 12 which reaches the maximum possible logarithm of odds ratio (LOD) score and fully co-segregates with the language impairment, consistent with an autosomal dominant, fully penetrant mode of inheritance. Whole genome analysis yielded no novel inherited copy number variants strengthening the case for a simple inheritance pattern. Several genes in this region of chromosome 12 which are potentially implicated in language impairment did not contain polymorphisms likely to be the causative mutation, which is as yet unknown.

Language is a unique human trait, rapidly mastered without explicit instruction, and is remarkably robust to external factors such as lack of language input and brain damage (Bishop & Mogford 1988). However, many children do have problems learning how to communicate. Disorders of communication such as dyslexia and specific language impairment (SLI) have long been known to have a large genetic component to their aetiology, reviewed in Bishop (2009). These disorders are likely caused by a combination of changes in many genes, as well as interactions with environmental factors, and show high co-morbidity with each other.

One of the most widely studied types of language impairment is a common, highly heritable (Bishop *et al.* 1995; Stromswold 2001) and profound impairment in language acquisition called SLI. Genome-wide scans of families presenting with SLI have uncovered three main areas of independently replicated linkage—chromosome 13q [SLI3, OMIM#607134 (Bartlett *et al.* 2002, 2004)], 16q (SLI1, OMIM#606711) and 19q (SLI2, OMIM#606712 (Falcaro *et al.* 2008; SLIC 2002, 2004)]. Two genes in SLI1, *ATP2C2* and *CMIP*, have recently been shown to modulate phonological short-term memory in language impairment (Newbury *et al.* 2009).

Although many cases of language impairment are multifactorial, mutations in a single gene on 7q31, *FOXP2*, have been shown to cause developmental verbal dyspraxia in a large pedigree, called the KE family (Lai *et al.* 2000, 2001), and a number of individual cases [reviewed in Fisher and Scharff (2009)]. *FOXP2* is a transcription factor important for modulating the plasticity of neural circuits in the developing brain (Groszer *et al.* 2008), and it is likely that downstream targets of *FOXP2* may have wide-ranging effects on language learning (Fisher & Scharff 2009), although it has not been directly implicated in SLI (Newbury *et al.* 2002).

Functionally there is evidence that children with language impairment have limitations in phonological short-term memory and also difficulties in processing rapidly changing sounds, called auditory processing, which is critical for phonetic distinctions in speech (Bishop *et al.* 1999, 2006; Tallal *et al.* 1996). Deficits in auditory processing have been shown to precede and predict language delay in infants (Benasich *et al.* 2002; Choudhury *et al.* 2007). Behavioral studies also indicate difficulties in the processing of phonological features which affect the segmentation of speech into meaningful words and phrases in cases with SLI (Penner *et al.* 2000; Weinert 1996). Further evidence has been provided by event-related brain potential (ERP) studies of infants at risk for SLI (Friedrich *et al.* 2009) and dyslexia; for a review see Friederici (2006).

Specifically, infants at risk for SLI deviate from age-matched controls in their discrimination ERP response for syllable length (Friedrich *et al.* 2004). The typical discrimination ERP response for auditory stimuli in adults is the so-called mismatch negativity (MMN). The MMN is elicited in a pre-attentive discrimination paradigm, in which subjects are presented with series of standard stimuli interspersed with a rarely occurring deviant stimulus. Upon registration of the change from standard to deviant stimulus, the brain responds with an enhanced negative-going peak in the ERP at 100–200 milliseconds after stimulus onset, and this is the MMN [for a review see Näätänen *et al.* (2001)]. The MMN is rather stable across age although a positive mismatch response has been reported for young infants (for a review see Friederici, 2006). A second negativity at a longer latency is sometimes observed in an MMN paradigm. This ERP response is referred to as either a late discrimination negativity (LDN) (Cheour *et al.* 2001) or a late MMN (Korpilahti *et al.* 1995). The LDN amplitude decreases as a function of age and is usually absent in the adult (Cheour *et al.* 2001). In children aged 4–7 years the LDN can still be observed but only in complex acoustic stimuli and word stimuli, and not for simple stimuli (Korpilahti *et al.* 2001). The LDN is thus considered as an index for a less mature mismatch response reflecting increased processing demands.

Here, we report the outcome of a study involving the NE family, which presents with language and literacy impairments spanning three generations. The family pedigree is given in Fig. 1. We tested the hypothesis that the language impairments in this family derive from a deficit in auditory discrimination using psychological and electrophysiological investigations. To uncover the genetic basis of the impairment we then carried out a whole genome linkage study, candidate gene sequencing and copy-number variation analysis.

**Figure 1:**
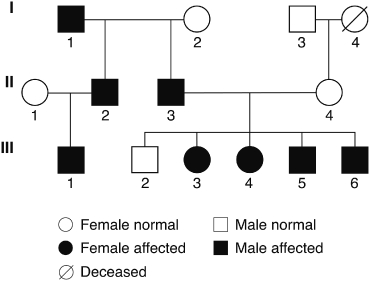
**Pedigree of the three-generational NE family.** All members indicated are native speakers of German, with the exception of II-1 whose first language is Portuguese and her second language is German, and III-1 who was brought up to be a bilingual. Circles indicate females and squares males. Black shaded figures indicate affected individuals.

## Methods

### NE family

The family under examination in the present study is a native German family affected by a language impairment which spans three generations. A genetic pedigree is given in Fig. 1. Written consent to participate in the study was obtained from all subjects or, in the case of minors, from the child's parents. All family members who are classed as ‘affected’ reported delays in their language development, as well as other features, indicating an auditory processing deficit, which is discussed below. Family members who are classed as ‘unaffected’ did not show any of these features. Table 1a gives information concerning age at initial recruitment, gender and clinical diagnosis of the individual family members based on the behavioral phenotype. The affected children III-3, III-4, III-5 and III-6 were initially referred to the clinic and were reported as having significant problems acquiring and using language. They either attended a specific school for children with language impairment, or received treatment to remediate their oral and written language problems. III-1 at the time of referral was too young for a clinical diagnosis but shows some problems with phonological awareness. III-3 and III-5 have partially compensated for the language delay. III-3, however, still reports difficulties in orthography (written language) and grammar and was also diagnosed as being dyslexic at school. The paternal grandfather I-1 was also diagnosed as dyslexic and reported having language problems similar to the children. His two sons, II-2 and II-3, are both reported as having dyslexia, and in addition II-2 has auditory attention problems. All family members have normal nonverbal IQ (NVIQ). The deficit of the affected members thus seems to be primarily located in the language domain with some consequent impact on literacy acquisition, and hence appears to be a form of language impairment. All family members were screened for hearing and auditory discrimination tasks (except III-1). Hearing was within the normal range for all family members (see audiograms in Supporting Information and Fig. S1). Psychoacoustic tests showed auditory discrimination deficits for tone duration in all affected family members tested, i.e. II-2, II-3, III-3, III-4, III-5 and III-6. These deficits were not present in the unaffected members, II-3, III-4 and III-6. For details of auditory discrimination abilities of duration, intensity and frequency, see Supporting Information (Fig. S2 and Table S2). All family members except for generation I participated in the nonword repetition (NWR) test and ERP experiment. All participated in the genetic testing. The study was approved by the Review Board of the Charité, Medical Faculty of the Humboldt University, Berlin.

**Table 1:** Information concerning (a) the NE Family and (b) the control individuals

(a) NE Family				
			Clinical Diagnosis	
Code	Sex	Age at ERP experiment	Affection	Affection details
I-1*	Male	69	Affected	Self-classified, also dyslexia
I-2*	Female	66	Unaffected	Self-classified
I-3*	Male	80	Unaffected	Self-classified
I-4*	Female	Deceased	Unaffected	Self-classified
II-1	Female	43	Unaffected	Bilingual Portuguese-German, native speaker of Portuguese
II-2	Male	38	Affected	Dyslexia, auditory attention problems
II-3	Male	38	Affected	Dyslexia
II-4	Female	39	Unaffected	
III-1	Male	4	Affected	Too young for clinical diagnosis of affection. Bilingual
III-2	Male	18	Unaffected	
III-3	Female	15	Affected	Late speech/language onset and development, but within normal range by age 6. Problems with reading and writing
III-4	Female	14	Affected	Very delayed speech and language development—at age 4 had attained the level of a 2-year old. Attended a special day-care and school and received speech and language therapy. Currently well compensated. However, speech is still slowed down, and marked by some grammatical errors (prepositions and case marking in German) and has persisting difficulties in understanding text and instructions
III-5	Male	6	Affected	Delayed onset of speech and language—very few words spoken by age 2:6. Received speech and language therapy at specialist day-care, now compensating well
III-6	Male	5	Affected	Distinct speech and fine motor deficit. Speech shows primarily phonological deficits (syllable structure and articulation), normal comprehension
(b) Control Individuals				
Group	Number	Sex	Age; Mean (SD)	Controls

*ERP experiment*				
Group 1	9	Male	5.6 (0.5)	III-1, III-5, III-6
Group 2	10	Female	14.5 (0.5)	III-3, III-4
Group 3	5	Male	18.2 (0.5)	III-2
Group 4	11	5 M, 6 F	M 38.0 (0.0) F 37.6 (1.4)	II-1, II-2, II-3, II-4
*NWR experiment*				
Children	27	16 M, 11 F	7.3 (0.8)	All in generation III
Adults	51	21 M, 30 F	33.0 (0.7)	All in generation II

Code: I to III indicates the three generations, roman numbers indicate different family members in one generation (see also Figure 1).

* means that these members did not participate in the NWR testing and the ERP experiment. M indicates males and F indicates females.

### Behavioral testing: NWR test

An NWR test evaluating phonological short-term memory was conducted. In this test participants are asked to immediately repeat a nonsense word such as ‘blonterstaping’. A German version of the test consisting of a series of 19 words which ranged in length from two to five syllables was created. All words conformed to the phonological requirements of German, and syllables with an independent meaning in German were avoided where possible. There was some variation in the relative ease of articulation of the words because up to two syllables per word could include a consonant cluster. The words were recorded by a native German-speaking woman who was requested during recording to locate word stress according to what felt natural for German. Typically, word stress fell on the penultimate syllable for two-, three- and four-syllable words and on the third syllable for five-syllable words.

The words were presented over headphones at a volume of 70 dB SPL. Subjects were awarded one point for each correctly articulated syllable (maximum score 64). A group of 51 normal adults (30 female) with mean age of 33.0 years (SD 0.7) who had no history of language impairment served as a control group for the adult NE family members. A group of 27 typically developing children (11 female) with a mean age of 7.3 years (SD 0.8) were tested as controls for the children of the NE family (Table 1b). For adults scores below 52 were below the 10th percentile, and for children scores below 35 were below the 10th percentile. Written consent was obtained from all subjects or, in the case of minors, from the child's parents.

### ERP experiment: phonological discrimination

#### Stimuli and procedure

In a passive oddball paradigm (standard: *P* = 5/6,deviant : *P* = 1/6), the discrimination of a short consonant-vowel (CV) syllable [ba] in a stream of long CV syllables [ba:] was examined. To compare the neural responses to the short syllable both as a deviant (in a stream of long syllables as standards) and as a standard stimulus, two experimental runs were included: a frequently occurring standard long syllable /ba:/ with a duration of 341 milliseconds was occasionally replaced by a deviant short syllable /ba/ with a duration of 202 milliseconds, and a standard short syllable /ba/ was replaced by a deviant long syllable /ba:/. The short syllable /ba/ was recorded from infant-directed speech, produced by a young mother who is a native speaker of German. After recording and digitization (44.1 kHz, 16 bit sampling rate) the vowel was digitally lengthened (starting at 30 milliseconds after syllable onset) to obtain the second syllable /ba:/ (see Supporting Information, Fig. S3). In each experimental run, an inter-stimulus interval (ISI) of 855 milliseconds (offset to onset) was used. Stimuli were presented via a loudspeaker with an intensity of 64 dB SPL and while subjects were watching an age-adequate self-selected video film presented visually at the centre of a screen placed in front of them.

#### ERP recording

The electroencephalography (EEG) was continuously recorded from silver–silver chloride electrodes F7, F3, FZ, F4, F8, C3, CZ, C4, FC3, FC4, T7, T8, P7, P3, PZ, P4, P8, CP5, CP6, O1 and O2 (according to the 10–20 International System) attached to an elastic electrode cap. The ERP electrodes were referenced to the left mastoid. Electrooculograms (EOG) were bipolar recorded from supraorbital and infraorbital electrodes on the right eye as well as from electrodes located lateral to the left and right eyes. Impedances were below 5 kΩ. The EEG was amplified with PORTI-32/MREFA (Twente Medical Systems, Oldenzaal, The Netherlands, with an input impedance of 10^12^Ω and an analogue first-order low-pass filter of 5 kHz). The EEG data were digitized on-line at a rate of 250 Hz (digital filter from DC to 67.5 Hz), and stored on hard disk. Further analyses were performed off-line. The data were digitally high-pass filtered with 0.4 Hz to remove drifts from the EEG while preserving most of the original signal. The EEG was algebraically re-referenced to the average of the two mastoids. Trials exceeding a standard deflection of 30 µV within a sliding window of 200 milliseconds at electrode CZ were rejected automatically. Epochs of 1200 milliseconds from stimulus onset were averaged according to a 200-milliseconds pre-stimulus baseline.

#### Controls in ERP experiment

Four groups of age-matched unaffected controls were tested. Group 1 included 9 children (all male) aged 5–6 years (mean: 5.6 years). This group was used a control group for III-1, III-5 and III-6. Group 2 included 10 teenagers (all female) aged 14–15 years (mean: 14.5 years). This group served as the controls for III-3 and III-4. Group 3 included 5 adolescents (all male) aged 18–19 years (mean: 18.2 years) who provided a control group for III-2. Group 4 included 11 adults (5 male; 6 female) aged 35–39 years (mean 37.8) [male 38 years, female 35–39 years (mean 37.6)] and who respectively served as control groups for II-1, II-2, II-3 and II-4 (Table 1b).

Written consent was obtained from all subjects or, in the case of minors, from the child's parents. Subjects older than 12 years were paid for their participation, younger children received a present of their choice. Children and teenagers were recruited through kindergartens and schools in Leipzig. Normal hearing was confirmed in all subjects by measuring pure-tone and white-noise-audiograms, by discrimination tests of tone intensity, frequency, duration and interaural phase, by temporal discrimination tasks [sinusoidal amplitude modulation (SAM)] and temporal sequencing tasks. All subjects were right-handed according to the Edinburgh Test (Oldfield 1971). All were native speakers of German.

#### Data analysis of controls

Statistical analyses were performed by calculating mean amplitudes for three time windows that were chosen after visual inspection of the averaged ERP plots and separate statistical analyses of consecutive windows of 50 milliseconds. For the lateral electrodes, a three-way analyses of variance (anova) for repeated measures were conducted with the within-subject variables: ‘*Discrimination’* (deviant stimulus vs. standard stimulus) ‘*Hemisphere’* (left vs. right hemisphere) and ‘*Region’* (anterior vs. posterior region). The subsets of electrodes used for hemispheres and regions for the analyses were: left: F7, F3, FC3, C3, CP5, P7, P3, O1; right: F8, F4, FC4, C4, CP6, P8, P4, O2; anterior: F7, F3, FC3, C3, C4, FC4, F4, F8; posterior: CP5, P7, P3, O1, O2, P4, P8, CP6. This yielded four scalp quadrants: left anterior: F7, F3, FC3, C3; right anterior: F8, F4, FC4, C4; left posterior: CP5, P7, P3, O1; right posterior: CP6, P8, P4, O2. For the midline electrodes the anova was performed including the within-subject variables ‘*Discrimination’* (deviant stimulus vs. standard stimulus) and ‘*Electrode*’ (Fz, Cz and Pz).

#### Data analysis of NE family members

The statistics for all members of the NE family included a calculation of whether the ERP pattern for individual family members would fall within or outside the normal range (min–max) of the mean amplitude derived from the age-matched control groups. The range was calculated for each control group and time window separately. Confidence intervals (CIs) were calculated only for time windows which showed a significant effect for the variable ‘*Discrimination*’ or any significant interaction between the variable ‘*Discrimination’* and one of the topographical variables (‘*Hemisphere*’ or *‘Region’*) in the global anova. As a first step to determine the range, the difference between the deviant and the standard (deviant condition minus standard condition) was calculated. In a second step, the mean amplitude for all frontal electrodes was calculated. Third, the product of the level of significance and the standard deviation was either subtracted or added to the mean amplitude of the relevant time window (e.g. range = mean amplitude ± (1.96 ^*^ standard deviation)). Corresponding average values were calculated for members of the NE family and compared to the range intervals for the control group.

### Genetic linkage analysis

A genome-wide scan for linkage to the language impairment segregating in members of the NE family was undertaken. Ten family members were included in the initial genome screen. They are I-1, I-2, I-3, II-3, II-4, III-2, III-3, III-4, III-5 and III-6. After contacting members of the extended family, II-1, II-2 and III-1, they were then included along with the original family members in a further, more detailed analysis focusing on chromosomes which had shown linkage in the initial screen.

Blood samples were collected from all available family members and used to generate lymphoblastoid cell lines using standard protocols. Genomic DNA was extracted from the cell lines using the DNeasy spin protocol (QIAGEN, Germantown, MD, USA).

Prior to commencing the genome scan, computer simulations in the programme SLINK (Ott 1989) were used to ascertain the maximum possible LOD (logarithm of odds ratio) score and power of the analysis to detect a major gene effect in this family. Given the inheritance pattern of the pedigree, the model used was of a fully penetrant, dominant disorder with a rare allele frequency of 0.001. The maximum LOD score expected with the 10 original family members was 1.5. The addition of II-1, II-2 and III-1 increased the maximum possible LOD to 2.2.

Individuals were genotyped for highly polymorphic dinucleotide repeat microsatellite markers. Approximately 300 markers from the ABI PRISM v2.5 MD10 panel, spaced at ∼20 cM (Haldane) across the genome, were genotyped in the initial analysis. Markers from the HD5 set or custom markers from the Généthon map (Dib *et al.* 1996) were used to increase the density to ∼10 cM (Haldane) on chromosomes 4 and 12 around the areas of suggestive linkage for the second round of analysis. Optimal PCR conditions for each marker were determined, and annealing temperatures ranged from 53 to 60°C, using 2 or 3 mm MgCl_2_ and 30–35 cycles of amplification with BioTaq kits (Bioline, London, UK). DNA from the same two control individuals were included on each plate to act as internal size controls. The fluorescent labeling of the primers with 6-FAM, HEX or NED phosphoramidites allowed pooling of products which was performed in a 4:3:9 µl ratio. Two microlitres of the pooled products were mixed with 8 µl Hi-Di/ROX-500 size standard mix (22 µl ROX in 1 ml HI-Di) (Applied Biosystems, Foster City, CA, USA) and subsequently separated and detected by the ABI 3700 sequencer (Applied Biosystems).

Allele calling and genotyping was performed in the GENESCAN v3.1/GENOTYPER v2.0 software (Applied Biosystems). PEDCHECK was used to detect Mendelian inheritance errors and GENEHUNTER v2.0 was employed for haplotype analysis (Kruglyak *et al.* 1996; O’Connell & Weeks 1998). Given the pedigree structure, the mode of inheritance was assumed dominant, with a fully penetrant, rare (0.001%) disease allele frequency. The MERLIN software package (Abecasis *et al.* 2002) was used to carry out parametric linkage analysis. This linkage analysis was conducted using the binary measure of affection status, and not for quantitative traits based on the ERP or NWR data. For chromosome X, MerlinMX was used, scoring hemizygous males as homozygotes (Abecasis *et al.* 2002).

### Candidate gene sequencing

Because of the large number of genes in the linkage region, six candidate genes with known relevant functions were selected for sequence analysis. They are *CNTN1*, *FOXJ2*, *GRIN2B*, *NELL2, NAB2* and *SRGAP1*. Sequencing was performed on DNA samples isolated from three of the most severely affected family members (II-3, III-4 and III-6). Coding exons from all known potential splice variants of the three genes were amplified by PCR using BioTaq kits (Bioline), supplementing the BioTaq in a 9:1 ratio with Pfu Turbo proofreading polymerase (Stratagene, Agilent Technologies, Santa Clara, CA, USA). Products were amplified using a ‘touch-down’ PCR programme, decreasing the annealing temperature from 67.5 to 61°C over 13 cycles, with a further 29 cycles at 61°C. All primer sequences can be found in Supporting Information, Table S3. PCR product clean-up was carried out using ExoI (NEB, Ipswich, MA, USA) and SAP (USB, part of Affymetrix, Santa Clara, CA, USA) with standard protocols, and sequencing reactions, using BigDye Terminator v3.1 Cycle Sequencing kits, were run on a 3730xl DNA Analyzer (Applied Biosystems). Base calling and quality assessment were carried out using the Contig Express programme from the Vector NTI package (Invitrogen Life Technologies, Carlsbad, CA, USA).

### Copy-number variation analysis using QuantiSNP

DNA samples from II-3, III-4 and III-6 were genotyped using the Illumina Infinium II Assay on the BeadArray 300K chip. This whole-genome assay amplifies and then fragments the DNA, before capturing the fragments on a BeadArray chip by hybridization to immobilized SNP-specific primers. Allelic specificity is then conferred by enzymatic base extension reactions, and products are subsequently stained with fluorescently labeled antibodies for differential allele detection. Details of the set protocol can be found at http://www.illumina.com. Preliminary analysis was carried out in Illumina's BeadStudio software.

QuantiSNP Version 1.1 (Colella *et al.* 2007) was used to detect the presence of copy-number variation (CNV) in the three family members. The following parameters were used: GC correction, EM iteration = 5, *L* value = 1000000, Max. copy number = 4.

## Results

### Behavioral testing: NWR test

German NWR test scores for each family member are displayed in Table 2. In its original English version this test has been shown to be reliably sensitive to detection of language impairment (Bishop *et al.* 1996) and linkage studies using the NWR test as a quantitative measure of SLI have been robustly replicated (SLIC 2002, 2004). From this data it is clear that those members who were diagnosed clinically as affected (see Fig. 1) perform most poorly on the NWR test—results shown in Table 2a vs.2b.

**Table 2:** Summary of performance on the NWR test

Code	Age at testing with NWR (years)	Score (No. syllables correct)	Control (mean and interquartile range, 25–75%)	Category of performance
(a) Affected members				
III-6	9.03	24		Affected
III-5	10.09	38	47	Borderline
III-1	6.10	30	(41–52)	Affected
III-4	18.01	35		Affected
III-3	19.00	44	57	Affected
II-2	39.05	53	(55–60)	Borderline
II-3	42.05	48		Affected
(b) Unaffected members				
III-2	23.04	59	57	Normal
II-4	44.06	56	(55–60)	Normal
II-1	43.09	56		Normal

Performance categories are defined as: Normal (performance is within the range observed for normal adults); Borderline (performance is two or three points below the ‘normal’ range); Affected (performance scores below the 10th percentile found in control subjects). Scores below the normal age range indicate ‘affected’ in NWR. Scores below 35 for 6–8-year-old children indicate a performance deficit, as do scores below 52 in adults of 18–48 years. Note that grandparents (I-1, I-2, I-3 and I-4) were not tested.

Analysis of the patterns of errors made by the affected family members shows that the number of errors increased with increasing length of nonsense word. There was little evidence that more errors were made because of simplification of consonant clusters. Indeed the reverse was often true, i.e. consonant clusters were created through the insertion of an extra consonant before the following vowel. This pattern of response is in contrast to unaffected family members whose errors were often independent of syllable length. In sum, these data suggest that phonological short-term memory rather than speech motor ability is affected in the NE family.

### ERP experiment: phonological discrimination

In testing the hypothesis that the NE family is affected with an auditory processing deficit, we assessed how family members discriminate between sounds of different duration by following on from the methodology of Friedrich *et al.* (2004). We applied an ERP paradigm based on eliciting a mismatch response which allowed evaluation of pre-attentive discrimination ability for sound duration. In this paradigm, subjects are presented with series of standard (same) stimuli interspersed with a rarely occurring deviant (other) stimulus. If the brain registers the change in stimulus from the standard to the deviant it responds to the deviant stimuli with an enhanced negative-going peak in the ERP at 100 to 200 milliseconds after stimulus onset. This is called the MMN. The response is elicited independent of the subject's attention to the stimulus [reviewed in Näätänen *et al.* (2001)], and so can reliably used in young children with short attention spans.

#### Controls

**Figure 2:**
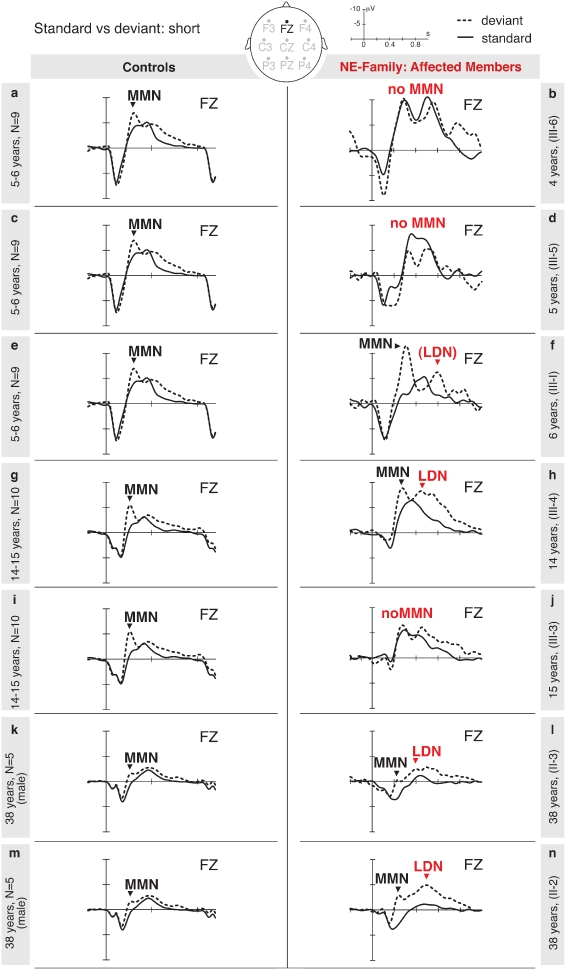

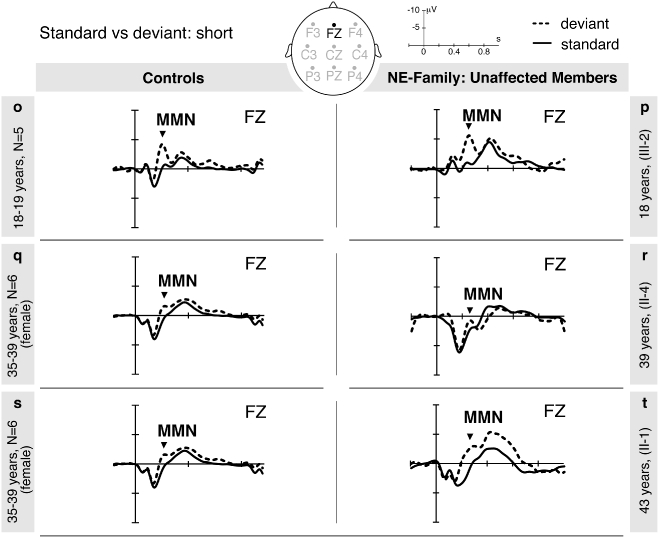
**(a) ERP data—affected members of the NE family; (b) ERP data—unaffected members of the NE family.** Averaged ERPs to short syllables for controls (left panel) in different age- and sex-matched groups and for members of the NE family (right panels). Solid lines are for the deviant and dotted lines for the standard stimulus. Negativity is plotted up.

In the control groups, as expected, the ERPs elicited by short syllable as deviants showed a clear early negativity, i.e. MMN at all ages (Fig. 2a and b, left panel). Note that for the CI analyses of the adult members of the NE family, separate analyses were run for males and females in the adult group to provide not only an age-matched, but also a gender-matched comparison. For brevity's sake, we will present the MMN data for the short deviant only. The statistical analyses of the mismatch responses for the different age-matched controls showed the following pattern: All groups showed an MMN for the short deviant, i.e. a main effect of ‘*Discrimination*’ was present in teenagers, adolescents and adults in the time window 175–275 milliseconds and in children between 250–350 milliseconds. Negativities in later time windows are observed for children and for teenagers, but within the control adult group only for males. The statistical analyses for the different age-matched control group are displayed in Table 3.

**Table 3:** Discrimination of vowel length in controls

Time windows (ms)	d*f*	175–275	250–350	400–500	500–700
**Children (5–6 years)**					
Discrimination	1, 8	1.61	54.58***	0.61	20.58**
Discrimination × Hem	1, 8	0.04	0.00	0.12	1.18
Discrimination × Reg	1, 8	0.08	4.95	4.32	0.64
Discrimination × Hem × Reg	1, 8	0.93	0.54	2.28	3.47
Midline	1, 8	1.91	73.58***	0.48	23.68***
Time window (ms)		175–275	300–400	500–700	
**Teenagers (14–15 years)**					
Discrimination	1, 9	142.38***	7.22*	8.55*	
Discrimination × Hem	1, 9	0.65	0.84	3.45	
Discrimination × Reg	1, 9	20.34**	0.60	2.58	
Discrimination × Hem × Reg	1, 9	0.05	3.35	0.90	
Midline	1, 9	75.91***	1.61	10.63**	
**Adolescents (18 years)**					
Discrimination	1, 4	19.79*	3.64	1.24	
Discrimination × Hem	1, 4	18.45*	1.28	0.07	
Discrimination × Reg	1, 4	16.33*	0.52	2.89	
Discrimination × Hem × Reg	1, 4	0.45	0.05	13.24*	
Midline	1, 4	25.68**	2.13	2.09	
**Adults—All (35–41 years)**					
Discrimination	1,10	26.28***	9.09*	7.16*	
Discrimination × Hem	1,10	0.00	0.00	0.00	
Discrimination × Reg	1,10	14.35**	0.01	2.33	
Discrimination × Hem × Reg	1,10	2.78	0.08	2.07	
Midline	1,10	32.67***	7.38*	4.72	
Discrimination × Gender	1, 9	0.63	0.64	10.88**	
**Adults—Males (36–38 years)**					
Discrimination	1, 4	8.92*	13.48*	68.34**	
Discrimination × Hem	1, 4	0.02	0.07	0.14	
Discrimination × Reg	1, 4	10.14*	0.48	8.51*	
Discrimination × Hem × Reg	1, 4	4.31	0.09	1.40	
Midline	1, 4	18.80*	8.39*	32.40**	
**Adults—Females (35–41 years)**					
Discrimination	1, 5	26.64**	1.93	0.21	
Discrimination × Hem	1, 5	0.03	0.44	0.56	
Discrimination × Reg	1, 5	4.67	1.62	0.22	
Discrimination × Hem × Reg	1, 5	0.28	0.00	0.78	
Midline	1, 5	22.22**	1.39	0.06	

Table displays *F*-values for main effect of Discrimination (difference between ERP responses to short deviant and long standard vowels) and interactions (×) with Hemisphere (Hem, left vs. right), Region (Reg, anterior vs. posterior) in normal controls. Midline refers to the analysis concluded over the midline electrodes. Time windows are relative to stimulus onset. Bold values indicate a negativity (mean amplitude of effect <0).

* *P*≤ 0.05;

** *P*≤ 0.01;

*** *P*≤ 0.001.

#### NE family

All members with the exception of the two youngest children, 4 year old III-1 and 5 year old III-6, provided a MMN response to changes in syllable duration (Fig. 2a and b, right panel). Similar to controls, this peak was observed in the MMN time window, i.e. 250–350 milliseconds for children and 175–275 milliseconds for the other members. In the affected members, however, this MMN was followed by a LDN between 500–700 milliseconds, which was very clearly enhanced in II-2, III-3, III-4 and III-5.

To statistically evaluate the ERP results for the NE-family members, we applied a CI analysis to determine whether the single subject ERP data would fall within or outside the range of the age-matched control group. A CI was calculated with a Bonferroni adjustment for each member, and compared to their age-matched control group (see Table 4a and b). The calculations showed the following: family members III-6, III-5 and III-3 deviate from their controls by showing no MMN. Family members III-4, II-3 and II-2 deviate from their controls by showing a LDN which is clearly enhanced compared to control traces. An enhanced negativity between 500 and 700 milliseconds was also visible for III-1, but statistically it fell within the normal range. Members III-1 and II-2 additionally showed an enhanced MMN falling outside the normal range. Thus, all affected members displayed an ERP pattern for duration discrimination which fell outside the normal range.

**Table 4:** Discrimination of vowel length in ERP experiment: NE family vs. controls

Time windows	MMN	LDN
**(a) Affected members**		
*Children*		
	**250–350 ms**	**500–700 ms**
Controls (5–6 y)	[−4.125, −0.607]	[−3.388, 0.250]
III-6	0.095*	−0.932
Controls (5–6 y)	[−4.125, −0.607]	[−3.388, 0.250]
III-5	−0.564*	−1.166
Controls (5–6 y)	[−4.125, −0.607]	[−3.388, 0.250]
III-1	−**5.059°**	−2.708
*Teenagers/Adults*		
	**175–275 ms**	**500–700 ms**
Controls (14–15 y)	[−5.163, −1.408]	[−3.019, 0.126]
III-4	−**3.550**	−**3.108+**
Controls (14–15 y)	[−5.163, −1.408]	[−3.019, 0.126]
III-3	−0.122*	−0.866
Controls: Male (36–38 y)	[−3.560, −1.597]	[−1.544, −0.748]
II-3	−2.112	−**1.547+**
Controls: Male (36–38 y)	[−3.560, −1.597]	[−1.544, −0.748]
II-2	−**3.701°**	−**2.023+**
**(b)Unaffected members**		
	**175–275 ms**	**500–700 ms**
Controls (18 y)	[−4.827, −1.147]	[−1.022, 0.294]
III-2	−2.681	−0.407
Controls: Female (35–41 y)	[−4.283, −0.476]	[−2.319, 0.172]
II-4	−0.889	0.233
Controls: Female (35–41 y)	[−4.283, −0.476]	[−2.319, 0.172]
II-1	−3.310	−2.196

The peak height range (min–max) in µV was calculated for the anterior electrodes using the following procedure: first, the difference was calculated by subtracting the standard condition from the deviant condition. Second, mean amplitudes were calculated for these time windows that showed significant effect in the anova analyses. Third, the final range was determined by adding or subtracting the product of the standard deviation and the level of the significance (5% or 1.96). Controls were matched for age and/or sex and the final range shown in square brackets before the result for the NE family member. Note that grandparents (I-1, I-2, I-3 and I-4) were not tested. Bold values indicate values of family members that do not fall into the respective range calculated for the control group:

* indicates absence of MMN

° indicates enhanced MMN, +indicates enhanced LDN.

### Linkage analysis

Microsatellite markers spread across the genome were genotyped in the NE family with the aim of uncovering the genetic basis of the auditory processing deficit and language impairment. Simulations indicated that linkage analysis of the binary measure of affection status using an autosomal dominant model would have the power to pinpoint a chromosomal region containing candidate genes harboring a causative mutation.

**Figure 3:**
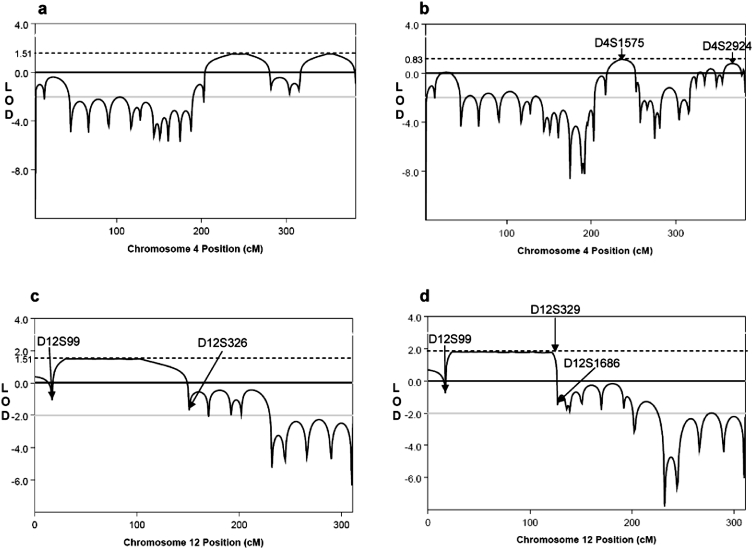
**Parametric linkage analysis plots from MERLIN.** All distances are in Haldane Centimorgans. Dotted lines indicate values of maximum LOD scores. a and b show linkage to chromosome 4 decreases in second wave analysis. (a) Plot with 10 original family members and markers 20 cM (Hal) apart. Maximum LOD score is 1.51. (b) Revised linkage plot with 10 cM (Hal) marker density in the distal arm of the chromosome, and including genotypes from II-1, II-2 and III-1. Arrows indicate positions of microsatellites D4S1575 and D2S2924, the phase of which could not be inferred. Maximum LOD score is 0.83. c and d show chromosome 12. The maximum LOD score increases on second wave analysis and critical region size decreases. (c) Plot with 10 original family members and markers 20 cM (Hal) apart. Maximum LOD score is 1.51. (d) Revised linkage plot with 10 cM (Hal) marker density in the proximal arm of the chromosome, and including genotypes from all 13 family members. Microsatellites, D12S99 and D12S329, indicate where breakpoints define the region of linkage. Maximum LOD score is 2.1.

Linkage analysis using the first 10 family members (excluding the branch containing II-1, II-2, III-1) yielded peaks which attained the maximum possible LOD score of 1.5 on chromosomes 4 and 12 (Fig. 3a and c). No other chromosome had a peak above 0 except chromosome 5, where the peak LOD of 0.6 was at the end of the chromosome and most likely because of an end effect, where the information content drops off because of the presence of no markers after the end of the chromosome.

In order to increase the power of the analysis, and to narrow the regions of linkage on chromosomes 4 and 12, three more family members were added for the second wave of the experiment, II-1, II-2 and III-1, along with many more markers at a higher density on the two chromosomes of interest. On addition of this fine mapping data from all 13 family members, the LOD score on chromosome 12 increased to 2.1 (max LOD calculated from SLINK 2.2) (Fig. 3d). The LOD score on chromosome 4 decreased with the second wave of analysis, but did not reduce to zero as the phase of alleles from D4S14745 and D4S2924 could not be ascertained in II-2 and III-1 because of homozygosity in the grandparent I-1 (Fig. 3b).

**Figure 4:**
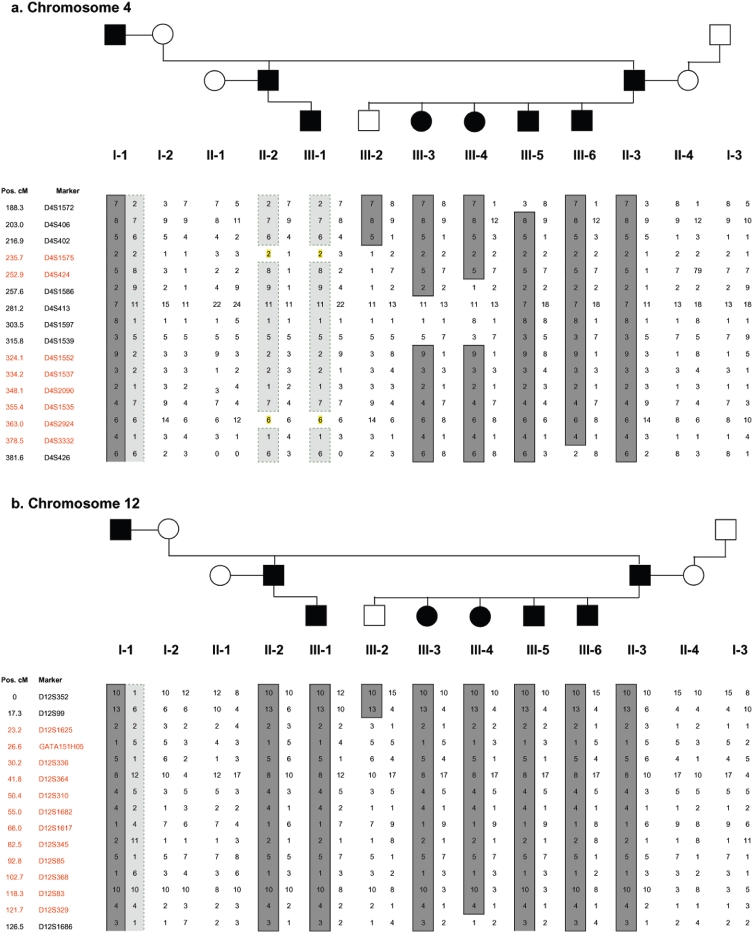
**Haplotype analysis of informative markers on chromosomes 4 and 12.** Paternal haplotypes on the left, maternal on the right. Affected individuals shaded in black in the pedigree. Alleles in dark gray are those shared by affected individuals in the initial screen. (a) Chromosome 4—indicates there is no segregation with the disorder in II-1 and III-1. It can be seen that although affected, II-1 and III-1 do not carry the ‘affected’ haplotype (dark grey) from I-1 showing lack of segregation with the disorder on chromosome 4. However, because of homozygosity of markers D4S1575 and D4S2924, highlighted, the phase of the alleles cannot be ascertained, meaning the LOD score can not be reduced to 0. (b) Chromosome 12—indicates continued and complete segregation of the risk alleles with the disorder. It can be seen that the affected chromosome (dark grey) is also inherited by II-1 and III-1, showing continued segregation with affection status.

We carried out haplotype reconstruction on the genotypes for chromosomes 4 and 12. It can be seen that III-1 does not inherit the ‘affected’ haplotype on chromosome 4 and that the continuing positive LOD scores are indeed only because of homozygosity and thus a lack of genetic information (Fig. 4a). However, Fig. 4b shows how the affected allele on chromosome 12 fully co-segregates with the disorder, and that the breakpoints in III-2 and III-4 define the critical linkage region.

The critical interval on chromosome 12, as defined by observed recombination events in individuals III-2 and III-4, spans a large 58.5-Mb region between markers D12S99 at 12p13.31 and D12S1686 at 12q14.3. This region contains almost 600 RefSeq genes (Feb 2009, hg19 Assembly).

### Candidate gene sequencing

The six candidate genes sequenced, *CNTN1*, *FOXJ2*, *GRIN2B*, NAB2, *NELL2* and *SRGAP1*, were selected based on their putative functions, and their relationships to genes potentially involved in the pathology of language impairments, auditory processing deficits, autism and dyslexia. *CNTN1* (Contactin 1, OMIM * 600016) is highly expressed in foetal brain. *CNTN1* is neural membrane protein which functions as a cell adhesion molecule and may be involved in forming axonal connections and in neuronal migration in the developing nervous system (Berglund & Ranscht 1994; Bizzoca *et al.* 2003). Another gene in the contactin family, *CNTNAP2,* is found to be mutated in the Amish population causing a rare neuronal migration disorder, cortical dysplasia-focal epilepsy (CDFE), resulting in language delay, seizures and autism (Strauss *et al.* 2006). *CNTNAP2* is also associated with autism (Alarcon *et al.* 2008; Arking *et al.* 2008; Rossi *et al.* 2008) and significantly correlated with NWR test scores in children with SLI (Vernes *et al.* 2008). *FOXJ2* (Forkhead box J2, GeneID 55810) is a member of the Forkhead Box transcription factor family. It is expressed very early in embryonic development, and has a wide tissue distribution, but a specific expression pattern in the brain (Wijchers *et al.* 2006). Over-expression of *FOXJ2* is embryonically lethal, most likely because of deregulation of downstream cell-adhesion genes like *Connexin-43* and *E-Cadherin* (Martin-de-Lara *et al.* 2008). Members of the FOX family are involved in many diverse developmental pathways, and mutations in *FOXP2* cause a distinct form of developmental verbal dyspraxia in the KE family, as previously discussed (Lai *et al.* 2001). *GRIN2B* (glutamate receptor, ionotropic, *N*-methyl d-aspartate 2B, OMIM *138252) is the NMDA receptor subunit 2B. The NMDA receptor channel is involved in long-term potentiation. This is an activity-dependent increase in the efficiency of synaptic transmission thought to underlie certain kinds of memory and learning. This gene had been associated with attention deficit hyperactivity disorder (ADHD) (Dorval *et al.* 2007), obsessive compulsive disorder (OCD) (Arnold *et al.* 2004, 2009) and bipolar disorder (Avramopoulos *et al.* 2007). *NAB2* (NGFIA-Binding Protein 2, OMIM*602381) is a binding protein which represses the activity of transcription factors *ERG1* and *ERG2* (Svaren *et al.* 1996). *NAB2* is most highly expressed in the brain and thymus and is necessary for Schwann cells to exit the cell cycle and coordinate the proper formation of myelin by acting as a co-repressor with NAB1 (Le *et al.* 2005; Mager *et al.* 2008). *NELL2* (NEL-like 2, OMIM * 602320) is a neuron-specific thrombospondin-1-like extracellular protein containing six epidermal growth factor-like domains, which plays a role in Ca^2+^-dependent intracellular events (Kuroda *et al.* 1999). *NELL2* functions during embryonic development as a neuronal survival and differentiation factor (Aihara *et al.* 2003). *NELL2* knockout mice show increased long-term potentiation and learning impairments in water mazes; thus, it appears that the *NELL2* also affects synaptic plasticity (Matsuyama *et al.* 2004, 2005). *SRGAP1* (SLIT-ROBO Rho GTPase activating protein 1, OMIM * 606523) is again highly expressed in foetal brain and is involved with *CDC24* in the pathway mediating the repulsive signaling of Robo and Slit proteins in neuronal migration (Bacon *et al.* 2009; Wong *et al.* 2001; Yao *et al.* 2008). *SRGAP3* is a candidate for severe X-linked mental retardation (Endris *et al.* 2002) and *ROBO1* is a candidate for dyslexia (Hannula-Jouppi *et al.* 2005).

No coding changes were found in *GRIN2B, FOXJ2, NAB2* or *NELL2*. A small number of changes were found in *SRGAP1* and *CNTN1* and are listed in Table 5; however all were synonymous and unlikely to alter gene expression. These changes are observed as common variants (SNPs) in European (CEPH) controls with similar frequencies. Only one change (*CNTN1* exon 12 c.1529hetC→T) was found in all three affected individuals sequenced. However, the T-allele is also found at a frequency of 70% in European controls.

**Table 5:** Coding changes observed in family members II-3, III-4 and III-6 from sequence analysis of genes *CNTN1*, *FOXJ2*, *GRIN2B, NAB2, NELL2* and *SRGAP1*

Gene	Exon	Base Change	rs number	Major allele freq	Effect on protein	Individuals with change
*SRGAP1*	18	c.2330hetT→C	rs789722	C/0.583	TCT→TCC p.758S	III-4, III-6
*CNTN1*	9	c.1127T→C	rs935105	T/0.842	AAT>AAC p.338N	II-3
	12	c.1529hetC→T	rs1056019	T/0.686	AAC>AAT p.472N	II-3, III-4, III-6

Base numbering from cDNA. All reference sequences obtained from UCSC Build 36.1. rs number and allele frequency obtained from the HapMap project.

### CNV analysis using QuantiSNP

In addition to the genome-wide linkage analysis, we also explored the possibility that the disorder was caused by CNV, leading to a loss or gain of genetic information which could directly alter gene expression and disrupt the language learning process. CNV is defined as a region of DNA 1 kb or larger which is present with a different number of copies to a reference genome, which usually carries two copies. Large-scale chromosomal rearrangements of >50 kb can also be detected by the SNP array used in this study. Classes of copy number variants include deletions, insertions and duplications [reviewed in Feuk *et al.* (2006)]. Increasingly, CNV has been shown to be important both for human diversity (Redon *et al.* 2006) as well as disease susceptibility, e.g. in autism and schizophrenia (Pagnamenta *et al.* 2009) as a significant proportion of copy-number variants can cover millions of base pairs of DNA and encompass many genes and their regulatory regions. Thus, if a gene is dosage sensitive, having the wrong number of copies is therefore likely to be deleterious. If a copy-number variant could be shown to be inherited along with the disorder in this family, and was not found in control populations, then it could be assessed as causative.

Results of the analysis defined six regions of interest, containing the same CNV in two or three of the individuals tested, with a Bayes cut-off of above 3 to be included in the group. These were deletions on chromosomes 2, 3 and 13, two duplications on chromosome 12, both within the region of linkage, and one on chromosome 22. The details of the variations are shown in Table 6. The duplication on chromosome 12 and deletion on chromosome 13 looked functionally most relevant, with the highest log Bayes factors and presence in II-3, III-4 and III-6. The regions were compared to the Database of Genomic Variants tracks (http://projects.tcag.ca/variation/) in the UCSC Genome Browser, Feb 2009(hg19) assembly (http://genome.ucsc.edu/), to ascertain if they were novel and therefore more likely to have a functional effect in this family. However, all of the CNVs seen in these individuals were located in regions of common structural variation, although often the exact breakpoints were novel. Therefore, it is unlikely that the language impairment seen in the NE family is because of large-scale structural variation.

**Table 6:** Results of QuantiSNP analysis of CNV in II-3, III-4 and III-6

Sample ID	Chr	Start (bp)	End (bp)	Length (bp)	Start (rsID)	End (rsID)	No. SNPs	Copy number	Log Bayes Factor
II-3	2	89772948	89932893	159945	rs2847840	rs842164	3	1	4.9518
III-4	2	89772948	89932893	159945	rs2847840	rs842164	3	1	11.2343
II-3	3	89489946	89499754	9808	rs9842599	rs870898	2	1	9.11556
III-6	3	89489946	89499754	9808	rs9842599	rs870898	2	1	10.1035
II-3	12	31101381	31298174	196793	rs244496	rs1025624	22	3	17.1712
III-6	12	31157554	31298174	140620	rs4931434	rs1025624	19	3	12.4023
III-4	12	31157554	31298174	140620	rs4931434	rs1025624	19	3	16.9481
II-3	12	36528296	36667312	139016	rs12306932	rs12368819	4	4	5.04966
III-6	12	36528296	36667312	139016	rs12306932	rs12368819	4	4	5.47972
III-4	12	36528296	36633905	105609	rs12306932	rs8186746	3	3	3.46073
II-3	13	22300993	22381716	80723	rs2335347	rs1887263	16	1	90.072
III-6	13	22320522	22381716	61194	rs11616753	rs1887263	15	1	63.7887
III-4	13	22294326	22381716	87390	rs1330919	rs1887263	17	1	87.5565
III-6	22	20654301	20892316	238015	rs732466	rs2330040	52	3	68.3679
III-4	22	20654301	20887622	233321	rs732466	rs5750720	51	3	82.1348

CNVs listed which occurred in two or more family members. Position, length, size (bp and number of SNPs involved) and type of CNV listed, as well as log-Bayes factor. A copy number of 1 indicates the deletion of one allele, and 3 or 4 the duplication of one or both alleles.

## Discussion

In this paper we have described the clinical picture of a three-generation German family with language impairment and consequential problems in literacy acquisition. We have carried out behavioral, electrophysiological and genetic testing on the family to elucidate the functional basis of this impairment.

The NWR test in its original English version has previously been found to be sensitive to the presence of language impairment in twin studies and subsequently has been shown to discriminate with a reasonable degree of sensitivity and specificity between parents of affected and unaffected children (Barry *et al.* 2007). The test is also useful as a diagnostic tool to identify people who have language impairment but have received speech and language therapy and are now well compensated, as is the case for the older children in the NE family (Bishop *et al.* 1996). The NWR test was administered to the family in a German version to ascertain if they also met one of the standardized criteria for language impairment. All affected members of the family scored below the normal range on the task with varying degrees of severity, and there was a clear correlation between the severity of the reported deficits in language and the NWR test scores. The numbers of errors made by the affected family members increased with the increasing numbers of syllables making up the nonwords and there was little evidence of an effect for difficulty of articulation. This pattern is consistent with Gathercole and Baddeley's hypothesis that deficits in NWR in SLI derive from deficits in phonological short-term memory (Gathercole & Baddeley 1990). It is interesting to contrast this data with that of the KE family who carry a mutation in *FOXP2*. Here poor results on the NWR test were primarily because of problems with articulation and not memory (Watkins *et al.* 2002).

The physiological reasons behind the family's poor language and literacy skills were investigated using electrophysiological experiments, where the data pointed toward difficulties in the discrimination of the duration of sounds. Discrimination of long vs. short CV syllables was investigated in an ERP experiment using a mismatch paradigm. The ERP pattern for all affected NE family members deviated from that of the average of the normal age-matched controls, although that of III-3 was borderline at the extreme low end of normal variation and could indicate a less severe version of the disorder. Controls in all age groups showed an MMN, which is the expected automatic response to a deviant stimulus. The most general observation for the affected members of the NE family is an absence of an MMN in the young family members and in the older members an enhanced second negativity between 500 and 700 milliseconds in addition to the MMN, namely a LDN (Cheour *et al.* 2001) not observed in controls. The relation of performance in the NWR test and the ERP pattern of the family members is indicated by a contingency analysis computing the relation of the categorization of ‘normal’ vs. ‘affected’ in each of the tests (see Table S4).

The amplitude of the LDN is known to decrease as a function of age in control subjects with only very small peaks in adults. In a language-impaired child who had received auditory discrimination training, the latency of the LDN peak also decreased (Cheour *et al.* 2001). In children aged 4–7 years, the LDN is not observed for simple stimuli such as tones but for complex, word-type stimuli (Korpilahti *et al.* 2001). Given the available literature, we suggest that the persistent LDN shown in the adults and teenagers of the NE family is indicative of a less mature mismatch response. The LDN reflects increased processing demands which then prolong the time it takes to discriminate between differences in the duration of CV syllables. The deviances in their ERP pattern for duration discrimination can be related to the observed deficits for tone duration in the psychoacoustic testing (Supporting Information). All affected family members performed below normal in the discrimination of tone duration and some also in the discrimination of frequency, although their hearing was within the normal range.

Deficits in the ability to process duration in general and syllable length in particular would render it difficult to discriminate between stressed and unstressed syllables and to identify the crucial parameters required for appropriately identifying intonational phrase boundaries which are important for marking word and sentence boundaries. Thus, deficits in duration discrimination as evidenced in the ERP data and the psychoacoustic testing would impact on correct segmentation of the speech input and hence on comprehension. From a developmental perspective, this would make the entrance into language more difficult, conceivably leading to delayed or impaired language development. Thus, the present data are in line with previous behavioral findings based on auditory processing deficits (Tallal *et al.* 1996). Such processing delays would significantly impact on a listener's ability to process multisyllabic words given that during normal speech perception 6-9 syllables must be processed per second. This immature processing ability would then clearly have an impact on NWR test performance, and also on the ability to deal with language input in normal conversational circumstances. Moreover, the data also agree with previous ERP studies showing a correlation of pathologically delayed ERP responses registered at the age of 2 months with behavioral language deficits measured at the age of 2–3 years (Friedrich *et al.* 2004). These findings, together with the combination of the ERP data, the psychoacoustic data and those of the NWR test in this family, present a very strong correlation between auditory discrimination abilities and language development.

A genome screen was undertaken in the NE family as the apparent mode of inheritance from the pedigree warranted a search for a single-gene mutation inherited in an autosomal dominant manner. Linkage analysis on all available family members identified a large region on chromosome 12 in which it is highly likely that the causal gene will be found. Although the size of the family and its structure meant that the maximum LOD score in this region did not reach the classical genome-wide significance level of 3.0, it is unlikely that this result represents a false positive, as the maximum possible LOD score for this pedigree was reached, and there is perfect segregation of the ‘affected’ haplotype on chromosome 12 in those family members who have language impairment. A lack of critical recombinations in the family on this chromosome means that the linkage region is sizeable and the number of candidates in the hundreds, and this cannot be resolved by typing more markers in the region. However, a search for any inherited copy-number variants in the family did not uncover any novel duplicated or deleted regions segregating with the disorder, strengthening the likelihood of a single gene effect. Sequence analysis of five functionally relevant candidate genes showed no coding changes at a frequency different to controls. Because of the multitude of other candidates in this region, the identification of the causative mutation will require narrowing the region of linkage. This could be achieved by genotyping any extended family member or performing genome screens on other families or individual cases presenting with a similar specific disorder. Because of the increasingly common use of high-throughput next generation sequencing technology, another approach would be to sequence a large number of the genes in this region to try and identify mutations which co-segregate with the impairment. Once more information becomes known about the physiological pathways leading to language impairment, as well as the downstream targets and interaction partners of *FOXP2*, *ATP2C2* and *CMIP* (Newbury *et al.* 2009) more candidate genes can also be sequenced on an individual basis.

In conclusion, although it is well known that most cases of language impairment appear to be multifactorial and are likely to involve changes in a multitude of genes as well as environmental factors, we have shown in this paper that there can also be cases which appear to be multigenerational and simply inherited, as in the NE family. We postulate that a single gene, located in the central region of chromosome 12, is mutated and underlies the auditory processing difficulties, leading to the subsequent language impairment in this family.

## Supporting Information

**Figure S1:** Audiogram of family members tested. Thresholds for audiogram against the tested frequency plotted in comparison with age-based standardized data. Data of the subjects II-4, II-3 and II-2 are compared with standardized age-based data ( age-group 30–39), data of subject II-1 are compared with age-based standardized data ( age-group 40–49) and the data of subjects III-3 and III-4 are compared with age-based standardized data (age-group 20–29 ) recorded by Bungert-Kahl *et al*. (2004) respectively. Gray box plots () show the 5% and 95% percentile and dark gray line and scatter plots () show the median and 95% confidence interval of the respective threshold for all recent tested normal hearing subjects of corresponding age group (II-2 was not able to hear test tones with a frequency of 16 000 Hz).

**Figure S2:** (a) Individual discrimination threshold values as *z*-scores referring to age-matched normative data (ordinate, logarithmic scale) for intensity (top panel), duration (middle panel) and frequency (lower panel) discrimination tests under monaural (left column) and dichotic (right column) stimulus conditions plotted separately for all family members (abscissa; arranged by increasing age). Dashed horizontal lines at 1.6 indicate the significance limit. Note that *z*-scores above 1.6 suggest impaired discrimination ability compared to age-matched normative data. All affected family members are displayed in color: II-3, II-2, III-3, III-4, III-5 and III-6. Open symbols for III-5 and III-6 indicate data from a separate second session conducted 4 years after the first session. (b) Cumulative distribution of discrimination threshold values (z-scores referring to age-matched normative data) of family members diagnosed with central auditory processing disorder for different discrimination tests (discrimination of frequency, intensity, and duration differences) under monaural and dichotic stimulus conditions compared to the respective values of one exemplary control group (15-year-old subjects). The values for the different discrimination tests are sorted and ordered by size (abscissa) between 0% and 100% (ordinate). Median, 10% and 90% percentiles are presented by dotted lines, and the z-score at significance threshold is indicated by the dashed vertical line at +1.6. Note that 35% and 53% of z-scores for duration and frequency discrimination, respectively, exceed the significance level.

**Figure S3:** Acoustic parameters of stimuli used in the ERP experiment. (a) Lengthened syllable /ba:/. (b) Short naturally produced syllable /ba/. (c) Represents the first 30 milliseconds of (a) and (b) which were identical for both stimuli.

**Table S1:** Psychoacoustic test parameters for audiogram and discrimination tests: *F* = frequency, Hz = Hertz, I = intensity, dB = decibel, *T* = time, ms = milliseconds.

**Table S2:** Performance in central auditory discrimination test for duration, frequency and intensity for mono = monoaural and dicho = dichotic presentation.

**Table S3:** Primer sequences used in candidate gene sequencing. Reverse primers are shown in the reverse complimentary direction. If an exon was too long to sequence from one product it was divided into two or more, and the products given the title ‘a’, ‘b’, ‘c’ etc. The different splice forms of *CNTN1*, *NELL2* and *SRGAP1* were identified from UCSC genome browser build 36.1 and use the annotation from this website (http://genome.ucsc.edu/).

**Table S4:** Relation between categorization of normal or affected/borderline in the NWR and the ERP pattern of each subject of the NE family. For classification criteria for NWR performance see Table 2 and for classification of ERP pattern see Table 4. Note that the present analysis only considers the absence of MMN and enhanced LDN as indicating affected (code: I-III indicates the three generations, roman numbers indicate different family members in one generation) (see also Figure 1).

As a service to our authors and readers, this journal provides supporting information supplied by the authors. Such materials are peer-reviewed and may be re-organized for online delivery, but are not copy-edited or typeset. Technical support issues arising from supporting information (other than missing files) should be addressed to the authors.

## References

[b1] Abecasis GR, Cherny SS, Cookson WO, Cardon LR (2002). Merlin–rapid analysis of dense genetic maps using sparse gene flow trees. Nat Genet.

[b2] Aihara K, Kuroda S, Kanayama N, Matsuyama S, Tanizawa K, Horie M (2003). A neuron-specific EGF family protein, NELL2, promotes survival of neurons through mitogen-activated protein kinases. Brain Res Mol Brain Res.

[b3] Alarcon M, Abrahams BS, Stone JL, Duvall JA, Perederiy JV, Bomar JM, Sebat J, Wigler M, Martin CL, Ledbetter DH, Nelson SF, Cantor RM, Geschwind DH (2008). Linkage, association, and gene-expression analyses identify CNTNAP2 as an autism-susceptibility gene. Am J Hum Genet.

[b4] Arking DE, Cutler DJ, Brune CW, Teslovich TM, West K, Ikeda M, Rea A, Guy M, Lin S, Cook EH, Chakravarti A (2008). A common genetic variant in the neurexin superfamily member CNTNAP2 increases familial risk of autism. Am J Hum Genet.

[b5] Arnold PD, Rosenberg DR, Mundo E, Tharmalingam S, Kennedy JL, Richter MA (2004). Association of a glutamate (NMDA) subunit receptor gene (GRIN2B) with obsessive-compulsive disorder: a preliminary study. Psychopharmacology (Berl).

[b6] Arnold PD, Macmaster FP, Richter MA, Hanna GL, Sicard T, Burroughs E, Mirza Y, Easter PC, Rose M, Kennedy JL, Rosenberg DR (2009). Glutamate receptor gene (GRIN2B) associated with reduced anterior cingulate glutamatergic concentration in pediatric obsessive-compulsive disorder. Psychiatry Res.

[b7] Avramopoulos D, Lasseter VK, Fallin MD, Wolyniec PS, Mcgrath JA, Nestadt G, Valle D, Pulver AE (2007). Stage II follow-up on a linkage scan for bipolar disorder in the Ashkenazim provides suggestive evidence for chromosome 12p and the GRIN2B gene. Genet Med.

[b8] Bacon C, Endris V, Rappold G (2009). Dynamic expression of the Slit-Robo GTPase activating protein genes during development of the murine nervous system. J Comp Neurol.

[b9] Barry JG, Yasin I, Bishop DV (2007). Heritable risk factors associated with language impairments. Genes Brain Behav.

[b10] Bartlett CW, Flax JF, Logue MW, Vieland VJ, Bassett AS, Tallal P, Brzustowicz LM (2002). A major susceptibility locus for specific language impairment is located on 13q21. Am J Hum Genet.

[b11] Bartlett CW, Flax JF, Logue MW, Smith BJ, Vieland VJ, Tallal P, Brzustowicz LM (2004). Examination of potential overlap in autism and language loci on chromosomes 2, 7, and 13 in two independent samples ascertained for specific language impairment. Hum Hered.

[b12] Benasich AA, Thomas JJ, Choudhury N, Leppanen PH (2002). The importance of rapid auditory processing abilities to early language development: evidence from converging methodologies. Dev Psychobiol.

[b13] Berglund EO, Ranscht B (1994). Molecular cloning and in situ localization of the human contactin gene (CNTN1) on chromosome 12q11-q12. Genomics.

[b14] Bishop DV (2009). Genes, cognition, and communication: insights from neurodevelopmental disorders. Ann N Y Acad Sci.

[b15] Bishop DVM, Mogford K (1988). Language Development in Exceptional Circumstances.

[b16] Bishop DV, North T, Donlan C (1995). Genetic basis of specific language impairment: evidence from a twin study. Dev Med Child Neurol.

[b17] Bishop DV, North T, Donlan C (1996). Nonword repetition as a behavioural marker for inherited language impairment: evidence from a twin study. J Child Psychol Psychiatry.

[b18] Bishop DV, Bishop SJ, Bright P, James C, Delaney T, Tallal P (1999). Different origin of auditory and phonological processing problems in children with language impairment: evidence from a twin study. J Speech Lang Hear Res.

[b19] Bishop DV, Adams CV, Norbury CF (2006). Distinct genetic influences on grammar and phonological short-term memory deficits: evidence from 6-year-old twins. Genes Brain Behav.

[b20] Bizzoca A, Virgintino D, Lorusso L, Buttiglione M, Yoshida L, Polizzi A, Tattoli M, Cagiano R, Rossi F, Kozlov S, Furley A, Gennarini G (2003). Transgenic mice expressing F3/contactin from the TAG-1 promoter exhibit developmentally regulated changes in the differentiation of cerebellar neurons. Development.

[b21] Cheour M, Korpilahti P, Martynova O, Lang AH (2001). Mismatch negativity and late discriminative negativity in investigating speech perception and learning in children and infants. Audiol Neurootol.

[b22] Choudhury N, Leppanen PH, Leevers HJ, Benasich AA (2007). Infant information processing and family history of specific language impairment: converging evidence for RAP deficits from two paradigms. Dev Sci.

[b23] Colella S, Yau C, Taylor JM, Mirza G, Butler H, Clouston P, Bassett AS, Seller A, Holmes CC, Ragoussis J (2007). QuantiSNP: an Objective Bayes Hidden-Markov Model to detect and accurately map copy number variation using SNP genotyping data. Nucleic Acids Res.

[b24] Dib C, Faure S, Fizames C, Samson D, Drouot N, Vignal A, Millasseau P, Marc S, Hazan J, Seboun E, Lathrop M, Gyapay G, Morissette J, Weissenbach J (1996). A comprehensive genetic map of the human genome based on 5,264 microsatellites. Nature.

[b25] Dorval KM, Wigg KG, Crosbie J, Tannock R, Kennedy JL, Ickowicz A, Pathare T, Malone M, Schachar R, Barr CL (2007). Association of the glutamate receptor subunit gene GRIN2B with attention-deficit/hyperactivity disorder. Genes Brain Behav.

[b26] Endris V, Wogatzky B, Leimer U, Bartsch D, Zatyka M, Latif F, Maher ER, Tariverdian G, Kirsch S, Karch D, Rappold GA (2002). The novel Rho-GTPase activating gene MEGAP/srGAP3 has a putative role in severe mental retardation. Proc Natl Acad Sci U S A.

[b27] Falcaro M, Pickles A, Newbury DF, Addis L, Banfield E, Fisher SE, Monaco AP, Simkin Z, Conti-Ramsden G (2008). Genetic and phenotypic effects of phonological short-term memory and grammatical morphology in specific language impairment. Genes Brain Behav.

[b28] Feuk L, Carson AR, Scherer SW (2006). Structural variation in the human genome. Nat Rev Genet.

[b29] Fisher SE, Scharff C (2009). FOXP2 as a molecular window into speech and language. Trends Genet.

[b30] Friederici AD (2006). The neural basis of language development and its impairment. Neuron.

[b31] Friedrich M, Weber C, Friederici AD (2004). Electrophysiological evidence for delayed mismatch response in infants at-risk for specific language impairment. Psychophysiology.

[b32] Friedrich M, Herold B, Friederici AD (2009). ERP correlates of processing native and non-native language word stress in infants with different language outcomes. Cortex.

[b33] Gathercole SE, Baddeley AD (1990). Phonological memory deficits in language disordered children: is there a causal connection?. J Mem Lang.

[b34] Groszer M, Keays DA, Deacon RM (2008). Impaired synaptic plasticity and motor learning in mice with a point mutation implicated in human speech deficits. Curr Biol.

[b35] Hannula-Jouppi K, Kaminen-Ahola N, Taipale M, Eklund R, Nopola-Hemmi J, Kaariainen H, Kere J (2005). The axon guidance receptor gene ROBO1 is a candidate gene for developmental dyslexia. PLoS Genet.

[b36] Korpilahti P, Lang AH, Aaltoneb O (1995). Is there a late-latency mismatch negativity (MMN) component?. Electroencephalogr Clin Neurophysiol.

[b37] Korpilahti P, Krause CM, Holopainen I, Lang AH (2001). Early and late mismatch negativity elicited by words and speech-like stimuli in children. Brain Lang.

[b38] Kruglyak L, Daly MJ, Reeve-Daly MP, Lander ES (1996). Parametric and nonparametric linkage analysis: a unified multipoint approach. Am J Hum Genet.

[b39] Kuroda S, Oyasu M, Kawakami M, Kanayama N, Tanizawa K, Saito N, Abe T, Matsuhashi S, Ting K (1999). Biochemical characterization and expression analysis of neural thrombospondin-1-like proteins NELL1 and NELL2. Biochem Biophys Res Commun.

[b40] Lai CS, Fisher SE, Hurst JA, Levy ER, Hodgson S, Fox M, Jeremiah S, Povey S, Jamison DC, Green ED, Vargha-Khadem F, Monaco AP (2000). The SPCH1 region on human 7q31: genomic characterization of the critical interval and localization of translocations associated with speech and language disorder. Am J Hum Genet.

[b41] Lai CS, Fisher SE, Hurst JA, Vargha-Khadem F, Monaco AP (2001). A forkhead-domain gene is mutated in a severe speech and language disorder. Nature.

[b42] Le N, Nagarajan R, Wang JY, Svaren J, Lapash C, Araki T, Schmidt RE, Milbrandt J (2005). Nab proteins are essential for peripheral nervous system myelination. Nat Neurosci.

[b43] Mager GM, Ward RM, Srinivasan R, Jang SW, Wrabetz L, Svaren J (2008). Active gene repression by the Egr2.NAB complex during peripheral nerve myelination.. J Biol Chem.

[b44] Martin-De-Lara F, Sanchez-Aparicio P, Arias De La Fuente C, Rey-Campos J (2008). Biological effects of FoxJ2 over-expression. Transgenic Res.

[b45] Matsuyama S, Aihara K, Nishino N, Takeda S, Tanizawa K, Kuroda S, Horie M (2004). Enhanced long-term potentiation in vivo in dentate gyrus of NELL2-deficient mice. Neuroreport.

[b46] Matsuyama S, Doe N, Kurihara N, Tanizawa K, Kuroda S, Iso H, Horie M (2005). Spatial learning of mice lacking a neuron-specific epidermal growth factor family protein, NELL2. J Pharmacol Sci.

[b47] Näätänen R, Tervaniemi M, Sussman E, Paavilainen P, Winkler I (2001). “Primitive intelligence” in the auditory cortex. Trends Neurosci.

[b48] Newbury DF, Bonora E, Lamb JA, Fisher SE, Lai CS, Baird G, Jannoun L, Slonims V, Stott CM, Merricks MJ, Bolton PF, Bailey AJ, Monaco AP (2002). FOXP2 is not a major susceptibility gene for autism or specific language impairment. Am J Hum Genet.

[b49] Newbury DF, Winchester L, Addis L (2009). CMIP and ATP2C2 modulate phonological short-term memory in language impairment. Am J Hum Genet.

[b50] O’connell JR, Weeks DE (1998). PedCheck: a program for identification of genotype incompatibilities in linkage analysis. Am J Hum Genet.

[b51] Oldfield RC (1971). The assessment and analysis of handedness: the Edinburgh inventory. Neuropsychologia.

[b52] Ott J (1989). Computer-simulation methods in human linkage analysis. Proc Natl Acad Sci U S A.

[b53] Pagnamenta AT, Wing K, Akha ES, Knight SJ, Bolte S, Schmotzer G, Duketis E, Poustka F, Klauck SM, Poustka A, Ragoussis J, Bailey AJ, Monaco AP (2009). A 15q13.3 microdeletion segregating with autism.. Eur J Hum Genet.

[b54] Penner Z, Weissenborn J, Wymann K, Weissenborn IJ, Höhle B (2000). On the prosody/lexicon interface in learning word order. A study of normally developing and language impaired children. Approaches to Bootstrapping: Phonological, Lexical, Syntactic, and Neurophysiological Aspects of Early Language Acquisition..

[b55] Redon R, Ishikawa S, Fitch KR (2006). Global variation in copy number in the human genome. Nature.

[b56] Rossi E, Verri AP, Patricelli MG, Destefani V, Ricca I, Vetro A, Ciccone R, Giorda R, Toniolo D, Maraschio P, Zuffardi O (2008). A 12Mb deletion at 7q33-q35 associated with autism spectrum disorders and primary amenorrhea. Eur J Med Genet.

[b57] SLIC (2002). A genomewide scan identifies two novel loci involved in specific language impairment. Am J Hum Genet.

[b58] SLIC (2004). Highly significant linkage to the SLI1 locus in an expanded sample of individuals affected by specific language impairment. Am J Hum Genet.

[b59] Strauss KA, Puffenberger EG, Huentelman MJ, Gottlieb S, Dobrin SE, Parod JM, Stephan DA, Morton DH (2006). Recessive symptomatic focal epilepsy and mutant contactin-associated protein-like 2. N Engl J Med.

[b60] Stromswold K (2001). The heritability of language: a review and meta analysis of twin adoption and linkage studies. Language.

[b61] Svaren J, Sevetson BR, Apel ED, Zimonjic DB, Popescu NC, Milbrandt J (1996). NAB2, a corepressor of NGFI-A (Egr-1) and Krox20, is induced by proliferative and differentiative stimuli. Mol Cell Biol.

[b62] Tallal P, Miller SL, Bedi G, Byma G, Wang X, Nagarajan SS, Schreiner C, Jenkins WM, Merzenich MM (1996). Language comprehension in language-learning impaired children improved with acoustically modified speech. Science.

[b63] Vernes SC, Newbury DF, Abrahams BS, Winchester L, Nicod J, Groszer M, Alarcon M, Oliver PL, Davies KE, Geschwind DH, Monaco AP, Fisher SE (2008). A functional genetic link between distinct developmental language disorders. N Engl J Med.

[b64] Watkins KE, Dronkers NF, Vargha-Khadem F (2002). Behavioural analysis of an inherited speech and language disorder: comparison with acquired aphasia. Brain.

[b65] Weinert S (1996). Prosody, short-term memory and speed of item identification: an empirical study on language processing deficits in specifically language impaired children. Sprache Kognition.

[b66] Wijchers PJ, Hoekman MF, Burbach JP, Smidt MP (2006). Identification of forkhead transcription factors in cortical and dopaminergic areas of the adult murine brain. Brain Res.

[b67] Wong K, Ren XR, Huang YZ, Xie Y, Liu G, Saito H, Tang H, Wen L, Brady-Kalnay SM, Mei L, Wu JY, Xiong WC, Rao Y (2001). Signal transduction in neuronal migration: roles of GTPase activating proteins and the small GTPase Cdc42 in the Slit-Robo pathway. Cell.

[b68] Yao Q, Jin WL, Wang Y, Ju G (2008). Regulated shuttling of Slit-Robo-GTPase activating proteins between nucleus and cytoplasm during brain development. Cell Mol Neurobiol.

