# Supplementary figures and images for: A locus for an auditory processing deficit and language impairment in an extended pedigree maps to 12p13.31-q14.3
Source: Genes Brain Behav. 2010 Aug;9(6):545–61. doi: 10.1111/j.1601-183X.2010.00583.x (PMC2948670; doi:10.1111/j.1601-183X.2010.00583.x)

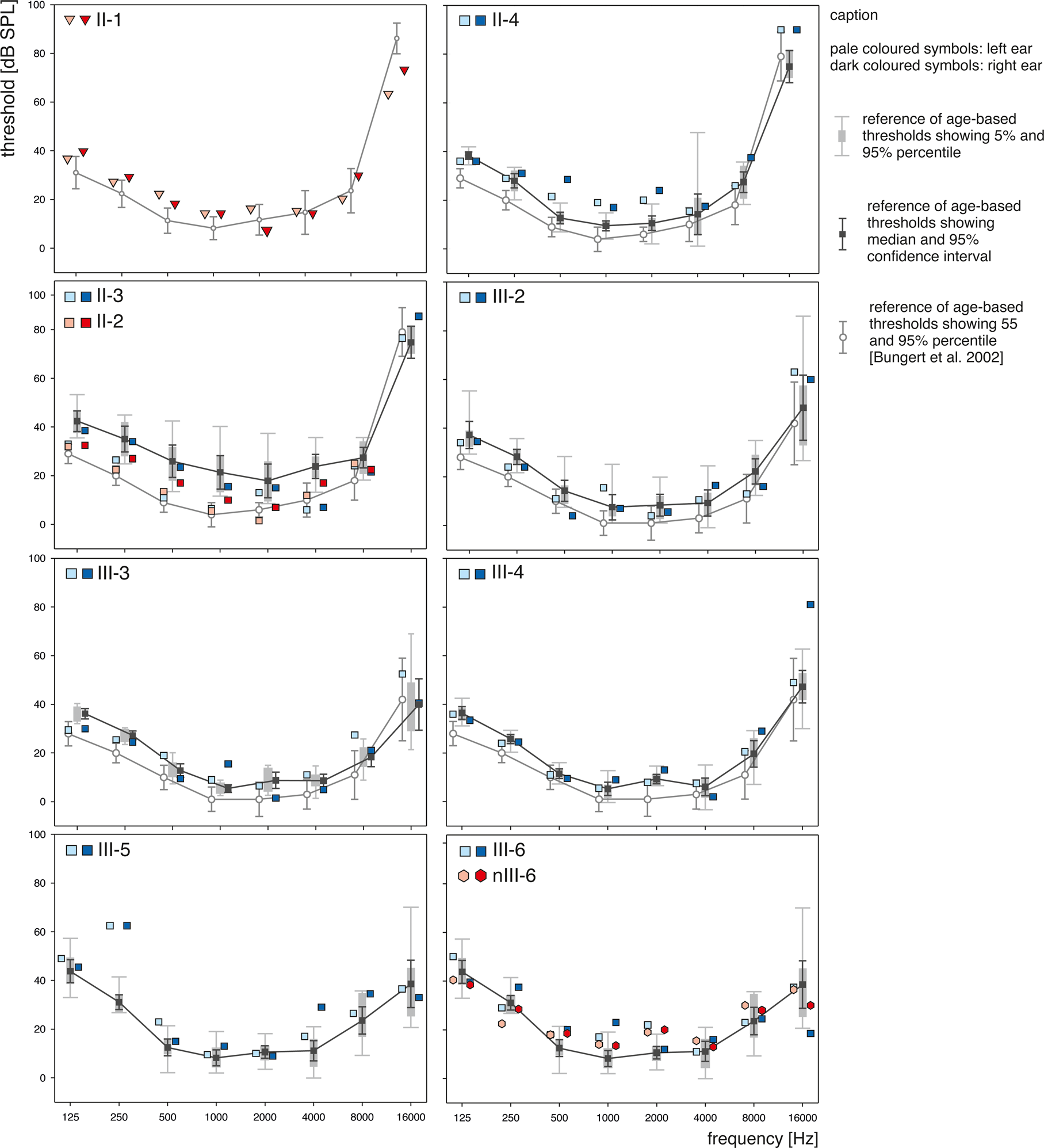

Supplement: Supplementary file 1 [file gbb0009-0545-SD1.tif]

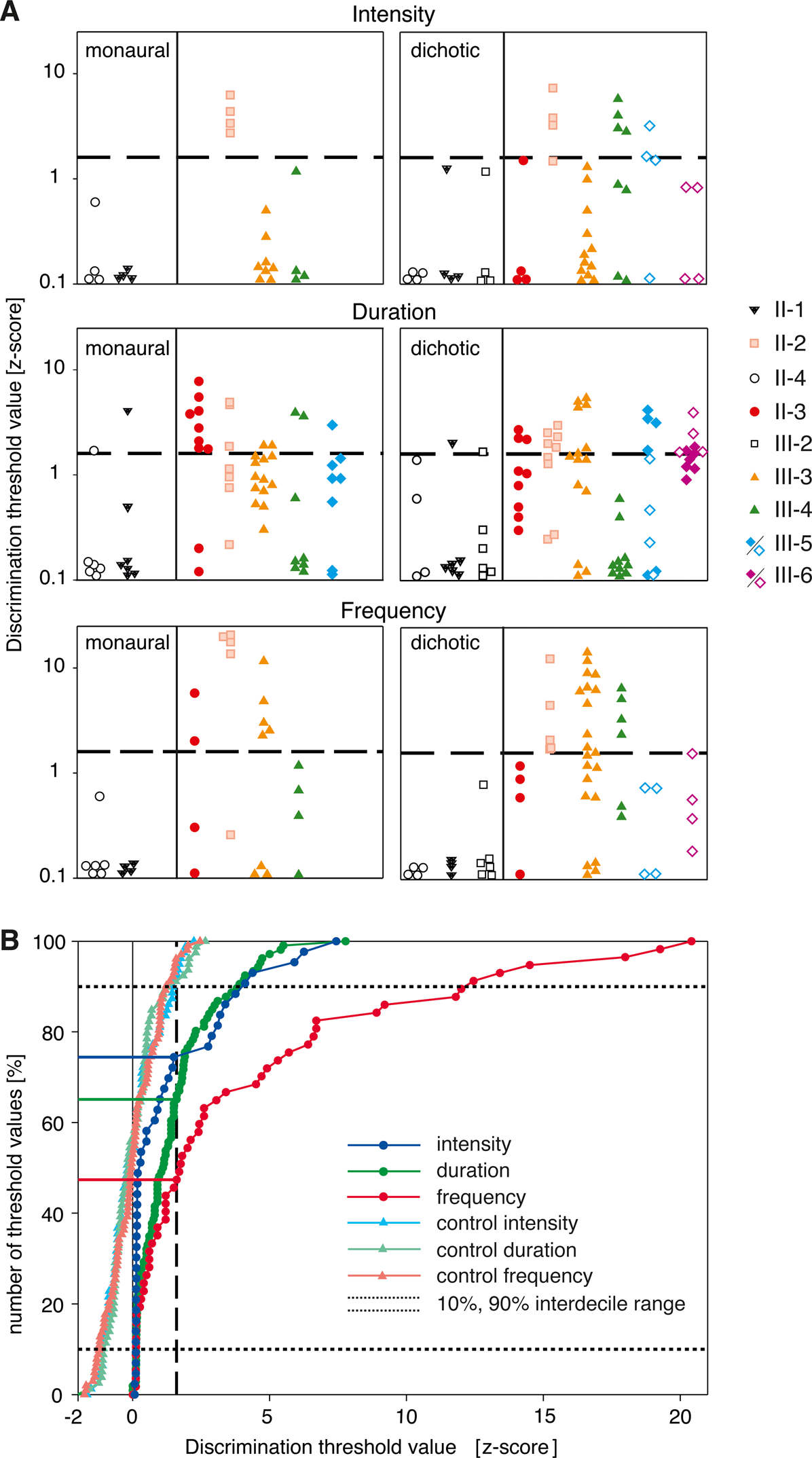

Supplement: Supplementary file 2 [file gbb0009-0545-SD2.tif]

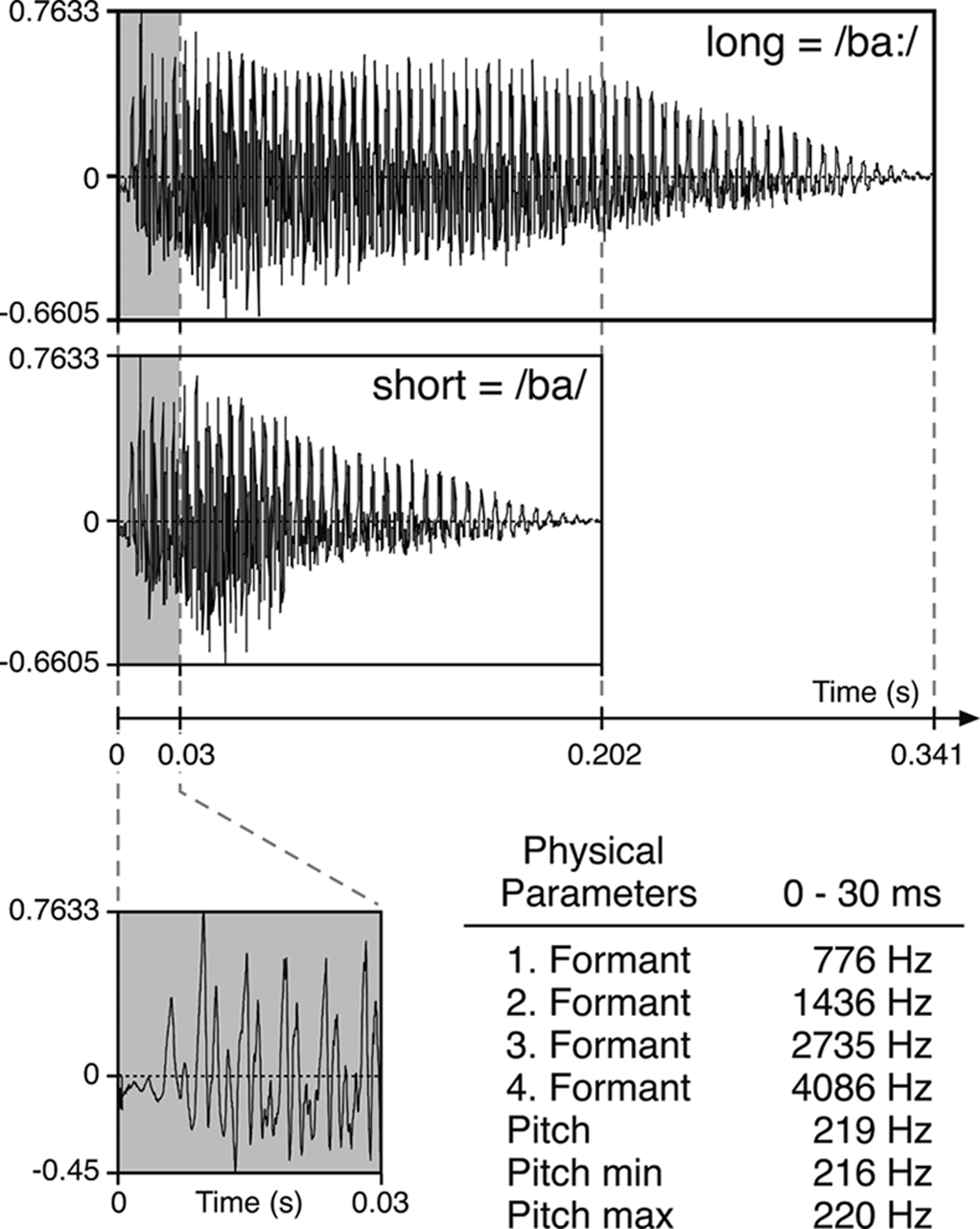

Supplement: Supplementary file 3 [file gbb0009-0545-SD3.tif]
