# Supplementary material for: A locus for an auditory processing deficit and language impairment in an extended pedigree maps to 12p13.31-q14.3
Source: Genes Brain Behav. 2010 Aug;9(6):545–61. doi: 10.1111/j.1601-183X.2010.00583.x (PMC2948670; doi:10.1111/j.1601-183X.2010.00583.x)
Supplement: Supplementary file 4 [file gbb0009-0545-SD4.doc]

**Supplementary Table 1:** Psychoacoustic test parameter overview

| Test | Test variable | Frequency of Test Signals, (kHz) | Signal duration  (ms) | Ramp  (ms) | Intensity | Initial Value | Mode of tests | Initial step size | Final step size |
| --- | --- | --- | --- | --- | --- | --- | --- | --- | --- |
| Audiogram | SPL | 0.125/0.25/0.5/1.0/2.0/ 4.0/8.0/16.0 | 250 | 10 | - | 60 dB SPL | additive | 10 dB | 2 dB |
| Frequency discrimination | ΔF, Hz | 0.25/0.5/1.0/2.0/ 4.0/8.0 | 250 | 10 | 30 dB SL | 1000 Hz | multiplicative | 2.0 | 1.1 |
| Intensity discrimination | ΔI, dB | 0.5/1.0/2.0/4.0 | 250 | 10 | 30 dB SL | 20 dB | additive | 5 dB | 2 dB |
| Duration discrimination | ΔT, ms | 0.5/1.0/2.0/4.0 | 250 | 10 | 30 dB SL | 100 ms | additive | 10 ms | 3 ms |

**Supplementary Table 2:** Central Auditory Discrimination Test

|  | | | | | | |
| --- | --- | --- | --- | --- | --- | --- |
| Family member | Duration | | Frequency | | Intensity | |
|  | mono | dicho | mono | dicho | mono | dicho |
| II-1 | + | + | + | + | (+) | + |
| II-2 | **-** | **-** | **-** | **-** | **-** | **-** |
| II-4 | + | + | + | + | + | + |
| II-3 | **-** | **-** | **-** | + | (+) | + |
| III-2 | (+) | + | (+) | + | (+) | + |
| III-3 | **-** | **-** | **-** | **-** | + | + |
| III-4 | **-** | **-** | + | **-** | + | **-** |
| III-5 | **-** | **-** | (+) | + | /. | **-** |
| III-6 | 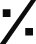 | **-** | (+) | + | (+) | + |
| III-1 | 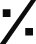 | 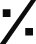 | 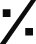 | 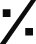 | 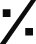 | 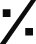 |

+ within normal range

**-** outside normal range

(+) not done as performance was normal in the dichotic condition which is taken to be

more difficult than the monaural condition


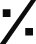
 not done as subjects did not want to undergo more testing

**Supplementary Table 3:** Primer sequences used in candidate gene sequencing

| ***CNTN1* - EXON** | **Forward Primer** | **Reverse Primer** | **Product Size bp** |
| --- | --- | --- | --- |
| rmm .ex1 | ccaactgccatagagctaaattc | aataaacacgcatatcaattatgttg | 187 |
| 2 | aagggccactactctcattcc | tgcttataatctcagaaagaccacc | 303 |
| 3 | ttcaatcttctcaaattttctttgac | acatctgtacaagttactagatgatg | 283 |
| 4 | gctccgtagagtttctaggtctc | tcctgattgttcttcttgctg | 304 |
| 5 | ggcttcaccaaaccactgag | cttgggactagagccaggtg | 427 |
| 6 | gcactgtttgtcttggctttc | tgtccttcctgaaaagtcagc | 464 |
| 7 | aagcttccatgttggagcag | acaagggttaaagtttcacacac | 327 |
| 8 | agtgtgtgaaactttaacccttg | tggaaatggctctaaataagatactg | 282 |
| 9 | gcctattactgagttcaggatgc | tcccaggactaccctgaaac | 335 |
| 10 | aagacaaccaaacccagacc | ttgtgtaggctcaataaacattttg | 279 |
| 11 | aacacagaagcaatgtttaggc | tgtccatcttagaactacaggatcttc | 362 |
| 12 | tccaaaacaaaatgtcaatgatg | tttcacttccaaattccaagaac | 482 |
| 13 | ttttcaaaagaatttcaggcaag | gaactttgcagacacatagg | 417 |
| 14 | tggtgttcattttgttagggg | gaatttgcaccagagttaccag | 314 |
| 15 | tggaattcactgattcattaccc | cctccaaatttttgtttttgag | 272 |
| 16 | tctccctgaaatagtaaaataaaaagg | cacatgcttttcagatgaatcac | 361 |
| 17 | aacatttgtggtttctgtggtg | gaaacaaaatggggtggaag | 243 |
| 18 | atccccatagcatttcctcc | gagggtgatgtttaatttggttc | 467 |
| 19 | ggtaaaagtgtgtgtgggaatg | ataggcccagttaggaacccc | 579 |
| 20 | aaatatgatgatcagccaacacc | gaaatcttgtgtggctgtaaagg | 435 |
| 21 | tgcagaattgagattggatatagtg | acccatcttgggtttcactg | 234 |
| 22 | tgcttaagtcacatctccttgg | tggacactggtgtatcaggc | 488 |
| 23 | atgcaatctcgccttagctg | gggttgaagggtgtcttctg | 221 |
| rmo. ex16 | aattgggcatccacactctc | ggtggggagaactgcagaag | 228 |

**Table S3 Cont.**

| ***GRIN2B* - EXON** | **Forward Primer** | **Reverse Primer** | **Product Size bp** |
| --- | --- | --- | --- |
| 1 | gtgagctgctctccataccc | gaatccctaccccaggtgag | 289 |
| 2a | agatggagttgggtttggtg | atgctctttgggtcggtctc | 386 |
| 2b | ttctcccaagttctggttgg | cacgctgtcaatgcaatctg | 510 |
| 3a | cccacttggttcaaaatctcc | tggccacttcaaagatgtagg | 403 |
| 3b | cctggacgatggagattctaag | catctccaccatcaatgtgc | 425 |
| 4 | tctccagccagctttctctc | agggacaaaagccaaaggac | 304 |
| 5 | tggccagtggtttctgattc | caaagctgactctcccatgc | 292 |
| 6 | ggcaaagggtaataaatgttgg | gaaaatggaaatggaaacagc | 285 |
| 7 | gccctcctgaatgttttaattg | ccatccatacgtccatttcc | 243 |
| 8 | tctgttctaaccctgagcactg | caaatgaggagtccagagatttg | 240 |
| 9 | accagccacaatggcttaac | ggctgagaacaggattgagg | 330 |
| 10 | ctcccgctctgtctctatctg | tgtgagaaacagatcaaggactc | 242 |
| 11 | cctccattggatttgtttgc | ggactggccatcagtagagg | 275 |
| 12 | tgctcctttcctgtctcctc | gcacagtgctaggctaagctg | 354 |
| 13a | gcctctctggagatctaacctg | ataggtgacggtgtgggttg | 654 |
| 13b | agtccaggtccatcagcaag | gttcttctcccaaggtgcag | 560 |
| 13c | atgactttaagcgcgactcc | gccctgtctttcaggcttac | 506 |
| 13d | gaagaaccggaacaaactgc | cccgtacccaccttaacctc | 613 |

| ***FOXJ2* - EXON** | **Forward Primer** | **Reverse Primer** | **Product Size bp** |
| --- | --- | --- | --- |
| 1 | tcgtgcctagacagtggaac | cccactagtgccagacaatg | 468 |
| 2 | cccccagtattttcgtctcc | tgaagcctaaacctaagccaag | 426 |
| 3 | tggaaaatgttctgctctgc | agccacaggactctggactg | 210 |
| 4 and 5 | accctgggaggttctgaatg | tctgaatctattcaccacccatc | 503 |
| 6 | ggttttattggtccctcagc | aaaatgtcaccccacttgttc | 325 |
| 7 | agggaaaatcccattttgtg | cccaccctcctctagatcac | 515 |
| 8 | tgcatgtagtcagcctggtatc | ccttccggttccagtttctac | 259 |
| 9 | caaggtttgcattgcttgc | ctccacctccatggcaac | 277 |
| 10 | tttcttccctgctttgttgag | caaaggaaagcaaagcaactc | 234 |
| 11 | gaacagcttcccatccactc | tccatgatgtagggacaggag | 434 |

**Table S3 Cont.**

| ***NAB2* - EXON** | **Forward Primer** | **Reverse Primer** | **Product Size bp** |
| --- | --- | --- | --- |
| 1 | cggagagagaagacgtggag | gaccccattcccaagtcc |  |
| 2 | tggtacccagtagggggact | agaggtaagcggctggagac | 994 |
| 3 | aaagcaagcgtctgatggat | catagcaggattccagtcca | 297 |
| 4 and 5 | ggttctgtgctctgccagtc | ctactcccccaccctcaatg | 397 |
| 6 | ggctggctatgtggttgag | tatccctcttcctcccacct | 362 |
| 7 | ctgttcccaaccacctctgt | tgggagtgtgttgtgtgagg | 474 |

| ***NELL2* - EXON** | **Forward Primer** | **Reverse Primer** | **Product Size bp** |
| --- | --- | --- | --- |
| A. ex1 | gaacctctccaccaggacac | tccaaggtgctaagttgatgg | 339 |
| 2 | cgcttgattccaagaacctc | tactttgggtccgggaaaag | 231 |
| 3 | tgcattttgatggagcttttc | cggaactgaaatcacagtgg | 226 |
| 4 | gagtctgaaactcagtatttatcagc | cggcttcccaaatcataaag | 400 |
| 5 and 6 | catctattccatcccgtagacag | tctactttttgaaactcattagaaagc | 511 |
| 7 and 8 | ttgaccattgaacataatttgg | gggaacatcaaggatttttaagc | 393 |
| 9 | tgctggaaggatgaacaatg | aagagtgggagcatctgagttc | 226 |
| 10 | tgaatggcctgttctgttctc | ttgcccagattaaaccaatg | 314 |
| 11 | tgtggaatgctgttacaagatg | caaaaatgtatttcaaggttgagc | 271 |
| 12 | gctggatctggaaacttcttaac | ttctacttcatgtcagttgtactagcc | 250 |
| 13 | ttgcattgtcatagtttcatttacc | tcaacttgacaacctgggaac | 272 |
| 14 | ccagaatattgcccactgttg | tttgctaatgttttgttgtatttatgg | 292 |
| 15 | tgcaataaccaagactgaagg | gaggaaggtagagatggatgg | 272 |
| 16 | tccaaggggataatgaaaagc | tgagagcccacttgaaacttg | 282 |
| 17 | tcccagtttcaaggatttgc | tgatatattcctcaagttcaagagaag | 314 |
| 18 | acaactggcctgattgcag | tcaaaactcatcaagccatatacac | 357 |
| 19 | aatgtgtgtgcgtgtgtgtg | tgggcctaaattttctttcc | 297 |
| 20 | tccatttcctcctccctctc | gcagctggcaaagtgctc | 320 |
| 21 | gatcatttactaatggcttaaaaattc | gccccagtaattttccttttg | 297 |
| B. ex1 | tctttggctgcttttattcg | tactttgggtccgggaaaag | 491 |
| C. ex1 | gtgggaagcttggagtaagg | ctgaagatgagaggcgactg | 346 |
| D. ex1 | ttggatgtgggatgtgagag | cacagctgtttgaaaattcagg | 282 |

**Table S3 Cont.**

| ***SRGAP1* - EXON** | **Forward Primer** | **Reverse Primer** | **Product Size bp** |
| --- | --- | --- | --- |
| srv. ex1 | tcgtgacttatatcttgcacatacc | aatccacaaaggtggcaaag | 346 |
| 2 | attggggcaactttgaaatg | ggcagttattggaaagcacac | 259 |
| 3 | ggcttgctgtctttctgtcg | tgtcactctggcttggaatg | 270 |
| 4 | ggttcccattcactctctaaatg | tgtggatatcagctattgcatc | 331 |
| 5 | aaaaatgactttgcattctttcc | ttttccaatcaacgaaatatgg | 252 |
| 6 | tttgcattcatttttgtatgtgg | cctctgcaaggacaggagag | 385 |
| 7 | cttctggaggtgggttgatg | agattactggtgtggcaggac | 193 |
| 8 | tctccctgtttgtgggattc | tagagtggcttgggaagtgg | 290 |
| 9 | tggcgtctagaagatcaaagtg | tccaggtcatcttcccatac | 530 |
| 10 | ccacatacactctgctgattcc | ttcagcattgaaatggttgc | 161 |
| 11 | ttgatttcccatgacttgagg | ttaaagccaagcatgagcac | 239 |
| 12 | ctctcccttgaggttaactgg | cccccatcacattgtctagg | 204 |
| 13 | aatgtgatgggggttttatcc | gggtgccacttcagtttacag | 258 |
| 14 | ttgttcagagaaaaagaagcactg | tgccttttccaggaagttagc | 181 |
| 15 | tgtggcaagtgaaaggactc | ggccttttgtcagagcattc | 403 |
| 16 | tcactttctttggagtttgctg | aattttcggtgggagaggag | 479 |
| 17 | cagcagtgcaagaacctgtc | tggcgctatcctagttcactg | 290 |
| 18 | accatgccttcctaacaacc | tggctggagtcctctattgg | 310 |
| 19 | taaaagaagggctttcatgc | aacagggagaaaacagaagagc | 459 |
| 20 | ccggtgtgataagctcttctg | cacacctcctacatcacacttagg | 449 |
| 21a | tactctgctgggccgtttac | tgcccgtgttaatttagctagtc | 651 |
| 21b | aggccataaagggaggtgac | gaaagaggcaagcatccaac | 565 |
| 21c | acaatgagcaccttcggttc | ggagaggacagttgggatttc | 723 |
| sru. ex1 | cgtgtgggagtacaactctgc | aggcacacaaaaggagcaag | 201 |
| sru. ex12 | ttgtttgatttctgccatgc | tttctgggaaagcaaagagc | 274 |
| srt. ex10 | aaccccagaggaagaggttg | cgcttcctcaaggttagcac | 412 |

**Supplementary Table 4:** Relationship between ERP and Nonword Repetition Data

|  |  | Nonword RepetitionWR | |
| --- | --- | --- | --- |
|  |  | normal | affected /  borderline |
| ERP | normal | III-2, II-4, II-1 | III-1 |
|  | affected / borderline |  | III-6, III-5, III-4,  III-3, II-3, II-2 |
